# Wandering Pneumonia

**DOI:** 10.31662/jmaj.2023-0063

**Published:** 2023-09-13

**Authors:** Naoya Fujita, Yosuke Ono

**Affiliations:** 1 Department of General Medicine, National Defense Medical College, Saitama, Japan

**Keywords:** Wandering pneumonia, IgG4-related lung disease, IgG4-related disease

A 74-year-old man was referred for chest X-ray abnormalities (Figure 1a). He had a history of hypopituitarism with pituitary stalk enlargement, soft tissues around the infrarenal and iliac arteries, and elevated serum IgG4 levels (150-250 mg/dL; reference range, 11-121 mg/dL), suggesting IgG4-related disease ^(1)^. He was treated with oral hydrocortisone and dexamethasone for hypopituitarism. Fine crackles were heard on chest auscultation. Computed tomography showed pulmonary ground-glass opacities with peribronchovascular and septal thickening. Because he was asymptomatic, he refused further investigation and underwent follow-up only. Dexamethasone was tapered and discontinued because of the exacerbation of diabetes mellitus, whereas the lung lesions progressed and the serum IgG4 levels increased. Subsequent chest X-rays at 868, 986, and 1,048 days after the first X-ray revealed spontaneous migration or disappearance of the shadows (Figure 1b-d), consistent with so-called “wandering pneumonia” ^(2)^. His serum IgG4 levels increased to 800-1,200 mg/dL during this time (Figure 2), but he had no respiratory manifestations even without immunosuppressive therapy. Thus, the imaging changes may have shown the formation of IgG4-related lung disease (IgG4RLD) over time ^(3)^. Although the long-term course of IgG4RLD is unclear, the present findings may be a feature of IgG4RLD.

**Figure 1.**
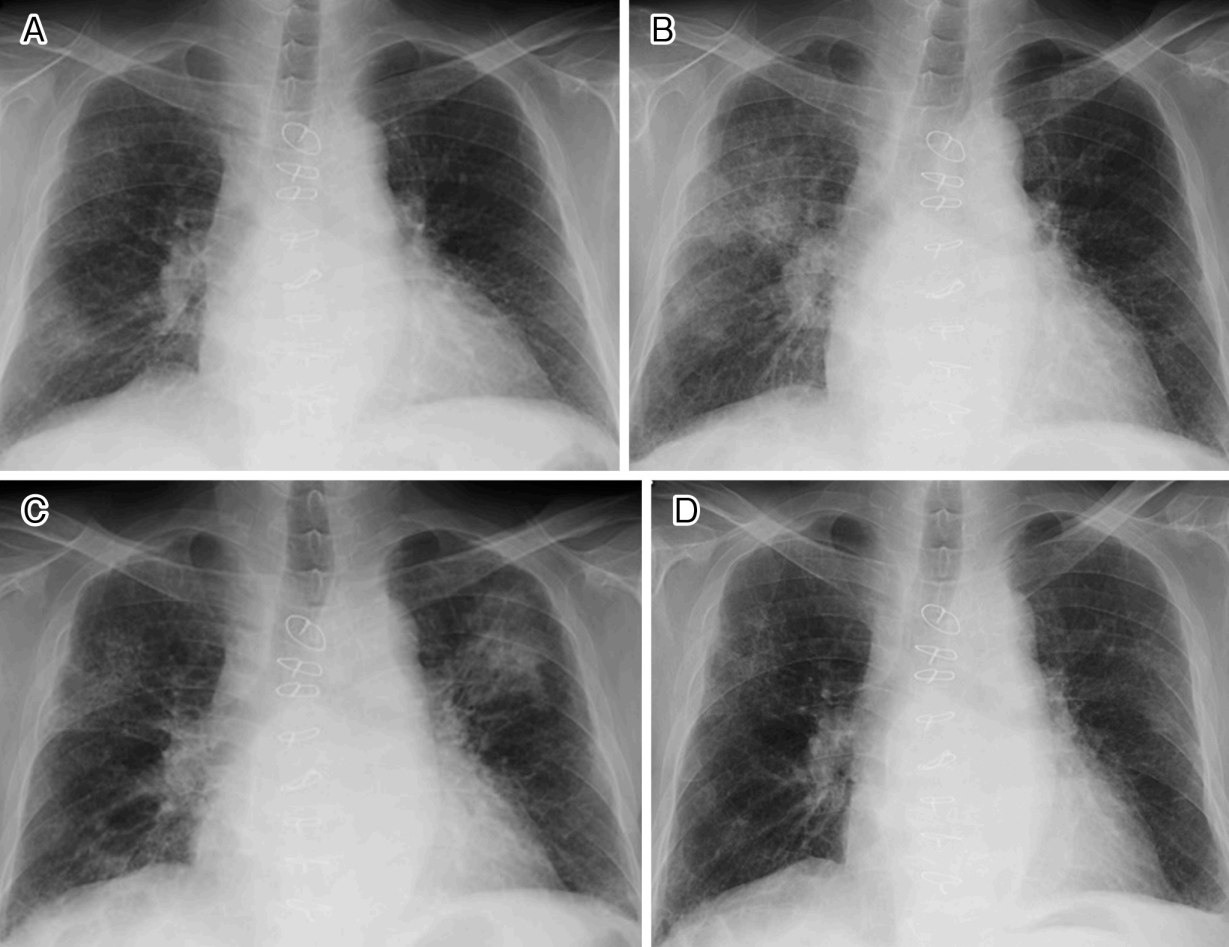
Abnormal shadow in the right lower lung field was observed in the first chest X-ray (1a). The shadow had migrated to the right upper lung on Day 868 (1b). The shadow had migrated in the left upper lung on Day 986 (1c). The shadow had almost disappeared on Day 1,048 (1d).

**Figure 2.**
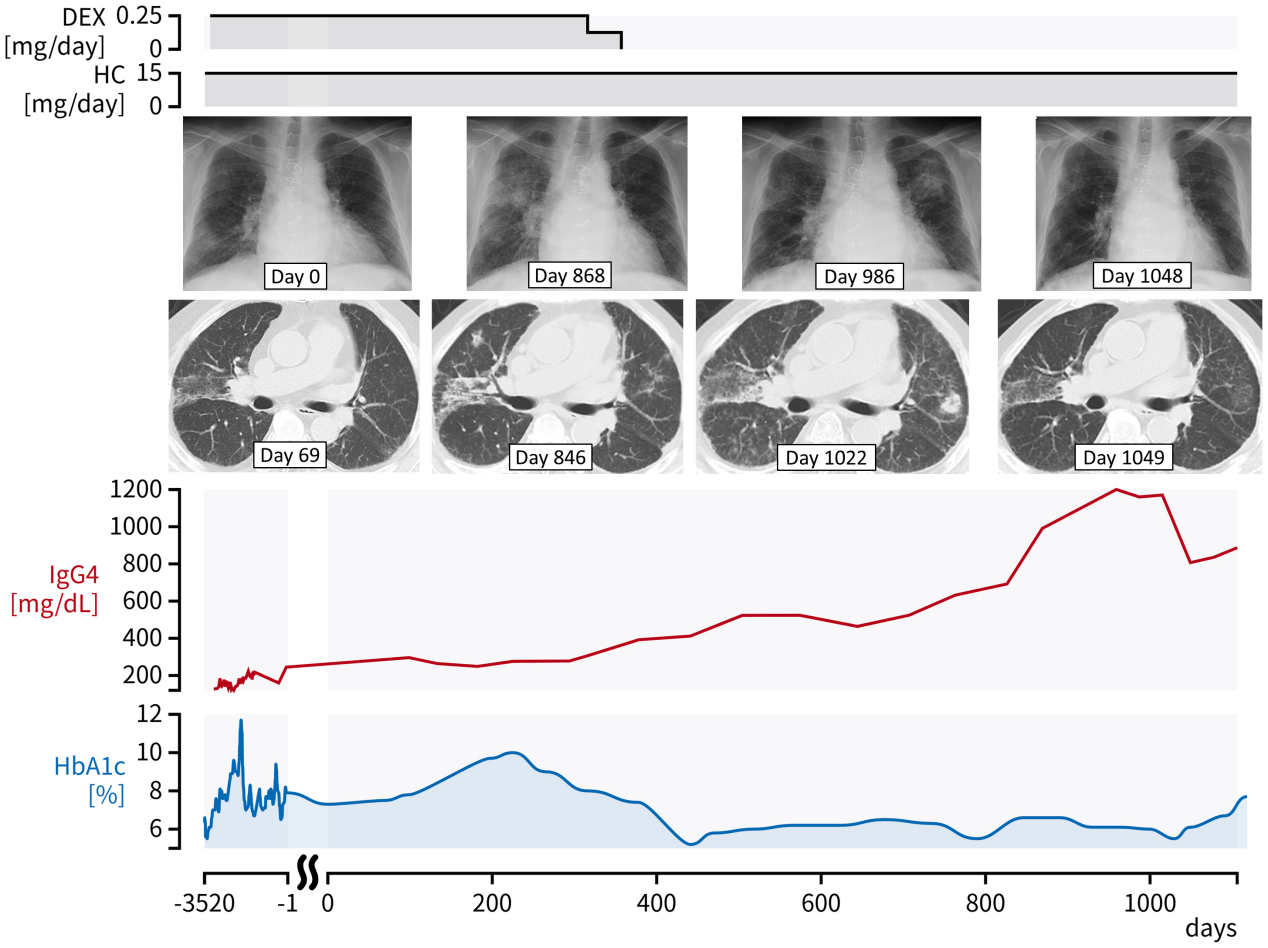
Clinical course of the patient. The patient had been treated with hydrocortisone and dexamethasone for hypopituitarism. Dexamethasone replacement was tapered and discontinued because of the exacerbation of diabetes mellitus, whereas the lung lesions progressed and the serum IgG4 levels increased up to the maximum of 1,200 mg/dL. He had been completely asymptomatic during this course. Day 0 is the day when chest X-ray abnormalities were recognized for the first time. DEX: dexamethasone, HbA1c: hemoglobin A1c, HC: hydrocortisone.

## Article Information

### Conflicts of Interest

None

### Sources of Funding

This work was supported by the Japan Medical Education Foundation (https://www.jmef.or.jp/). The funders played no role in the preparation of the manuscript or the decision to publish.

### Author Contributions

NF wrote the first draft of the manuscript and contributed to patient care. YO revised and organized the manuscript.

### Informed Consent

The patient provided written informed consent for the publication of the manuscript and the clinical images.

### Approval by Institutional Review Board (IRB)

This study did not require IRB approval.
